# Congenital coronary anomalies detected by coronary computed tomography compared to invasive coronary angiography

**DOI:** 10.1186/1471-2261-14-81

**Published:** 2014-07-08

**Authors:** Jelena R Ghadri, Egle Kazakauskaite, Stefanie Braunschweig, Irene A Burger, Michelle Frank, Michael Fiechter, Catherine Gebhard, Tobias A Fuchs, Christian Templin, Oliver Gaemperli, Thomas F Lüscher, Christian Schmied, Philipp A Kaufmann

**Affiliations:** 1 Department of Cardiology, University Hospital Zurich, Zurich, Switzerland; 2 Departement of Nuclear Medicine, Cardiac Imaging University Hospital Zurich, Ramistrasse 100, NUK C 40, Zurich CH-8091, Switzerland; 3 Hospital of Lithuanian University of Health Sciences Kaunas Clinics, Kaunas, Lithuania; 4 Zurich Center of Integrative Human Physiology (ZIHP) University of Zurich, Zurich, Switzerland

**Keywords:** Coronary anomalies, Computed coronary tomography angiography, Invasive coronary angiography

## Abstract

**Background:**

As coronary computed tomography angiography (CCTA) has emerged as a non-invasive alternative for evaluation of coronary anatomy with a lower referral threshold than invasive coronary angiography (ICA), the prevalence of coronary anomalies in CCTA may more closely reflect the true prevalence in the general population. Morphological features of coronary anomalies can be evaluated more precisely by CCTA than by ICA, which might lead to a higher identification of congenital coronary anomalies in CCTA compared to ICA.

To evaluate the incidence, clinical and morphological features of the anatomy of patients with coronary anomalies detected either by coronary computed tomography angiography (CCTA) with prospective ECG-triggering or invasive coronary angiography (ICA).

**Methods:**

Consecutive patients underwent 64-slice CCTA (n = 1′759) with prospective ECG-triggering or ICA (n = 9′782) and coronary anatomy was evaluated for identification of coronary anomalies to predefined criteria (origin, course and termination) according to international recommendations.

**Results:**

The prevalence of coronary anomalies was 7.9% (n = 138) in CCTA and 2.1% in ICA (n = 203; p < 0.01). The most commonly coronary anomaly detected by CCTA was myocardial bridging 42.8% (n = 59) vs. 21.2% (n = 43); p < 0.01, while with ICA an absent left main trunk was the most observed anomaly 36.0% (n = 73; p < 0.01). In 9.4% (n = 13) of identified coronary anomalies in CCTA 9.4% were potentially serious coronary anaomalies, defined as a course of the coronary artery between aorta and pulmonary artery were identified.

**Conclusion:**

The prevalence of coronary anomalies is substantially higher with CCTA than ICA even after exclusion of patients with myocardial bridging which is more frequently found with CCTA. This suggests that the true prevalence of coronary anomalies in the general population may have been underestimated based on ICA.

## Background

Coronary anomalies are defined as morphological features occurring in less than 1% [1,2] of an unselected population affecting the origin, course or termination of a coronary vessel [3]. Based on extrapolations of findings obtained during invasive coronary angiography (ICA) using this definition, the prevalence of any kind of coronary anomaly has been estimated at about 5.6% for the general population [3]. Better knowledge and early detection of coronary anomalies seems pertinent in view of the fact that they represent the underlying disease in approximately 19% of sudden cardiac death (SCD) in young athletes [4]. So far, ICA has been considered the first–line method for the assessment of coronary anomalies. Most commonly, coronary anomalies have been detected incidentally during the evaluation of patients with suspected coronary artery disease (CAD). In recent years, coronary computed tomography angiography (CCTA) has emerged as a non-invasive alternative for evaluation of coronary anatomy [5], particularly after introduction of modern protocols [6-8] allowing to perform CCTA with a radiation dose substantially lower than that of ICA [9]. CCTA has therefore been recommended as first-line method for the assessment of known or suspected coronary anomalies [10]. The availability of low dose scan protocols has lowered the threshold for referrals to CCTA and is gradually changing the evaluation strategy in many patients with low pre-test probability for CAD. Thus, the prevalence of coronary anomalies in CCTA may more closely reflect their true prevalence in the general population [11].

The aim of the present study was to compare the prevalence and anatomical characteristics of coronary anomalies in a large consecutive population undergoing either CCTA or ICA for different indications.

## Methods

### Selection of subjects

The prevalence of coronary anomalies in both diagnostic approaches (ICA and CCTA) was assessed retrospectively from the CCTA and angiography registry at the University Hospital Zurich. ICA or CCTA were performed due to suspicion of coronary anomaly or coronary heart disease as substantiated by symptoms such as angina pectoris or dyspnea.

We included all consecutive patients undergoing CCTA from February 2007 to October 2011 or ICA from August 2005 to July 2009. The reports of these patients were reviewed for coronary anomalies according to the definition suggested by Angelini et al. [3]. Cardiovascular risk factors and symptoms were obtained from medical records from our hospital based software system. The need for written informed consent was waived by the institutional review board (local ethics committee) due to the retrospective nature of the study with sole clinical data collection.

### CT data acquisition and post-processing

All scans were performed on a 64-slice CT scanner (LightSpeed VCT, GE Healthcare) with prospective ECG-triggering allowing acquisition of low dose CCTA as previously reported [7].

Briefly, intravenous metoprolol (5 to 20 mg) (Beloc, AstraZeneca, London, UK) was administered to achieve a target heart rate below 65 b.p.m before scanning. A single dose of 2.5 mg isosorbiddinitrate sublingual (Isoket, Schwarz Pharma, Monheim, Germany) was also applied prior to the scan. All patients were carefully monitored during the examination to assure that breathing commands were adequately followed [12]. All images were transferred to an external workstation (AW 4.4, GE Healthcare) for image reconstruction and evaluation.

In all patients in which a coronary anomaly, origin, course and/or termination of the coronary arteries had been originally reported, the angiograms were reassessed by two physicians experienced in cardiac computed tomography imaging.

### Invasive coronary angiography

ICA was performed according to standard techniques on an Allura 9 and an Allura XPER FD10/10 (Philips Medical Systems) catheterization system by experienced interventional cardiologists. Two experienced observers evaluated the coronary arteries based on the results of the invasive assessments.

### Effective radiation dose estimation

Values for radiation dose were estimated for CCTA as the product of the dose length product (DLP) × a conversion coefficient for the chest (k = 0.014 mSv/mGy × cm) as previously suggested [13,14]. Similarly, for ICA radiation dose was estimated as the product of the dose-area product (DAP) of the diagnostic coronary scenes x conversion factor for chest (k = 0.22 (mSv/mGy × cm^2^) for ICA based on the National Radiological Protection Board tables [15].

### Statistical analysis

Categorical variables were presented with absolute and relative frequencies (%), and continuous variables with mean ± SD. For between group comparisons, unpaired t test were used for parametric data, and Mann–Whitney tests for nonparametric data. A Person X^2^ or Fisher exact test was performed where appropriate. A *P* value <0.05 was considered to indicate a significant difference. All statistical data were analysed with SPSS software (version 19.0, SPSS Inc., Chicago, Illinois).

## Results

### Study population

From February 2007 to October 2011 CCTA was performed in 1′759 patients revealing 138 coronary anomalies, resulting in a prevalence of 7.9%. From August 2005 to July 2009 ICA was performed in 9′732 patients and 203 anomalies were found, corresponding to a prevalence of 2.1%.

Patients with coronary anomalies identified with coronary angiography were older compared to those in whom such findings were detected by CCTA. Coronary anomalies tended to occur slightly more often in male (64.5% by CCTA and 70.0% by ICA; p = n.s.) than in female patients (Table 1). Among the different risk factors, hypertension was most often observed in both patient groups (CT 39.3% and ICA 62.1% (p < 0.01); Table 1). Analysis of the symptoms of both groups revealed that typical chest pain (52.2%; p < 0.01) was more common in ICA group, while 47.8% of patients with anomalies from CCTA demonstrated no symptoms (p < 0.01).

**Table 1:** Patient characteristics

	**CCTA (n=1759)**	**ICA (n=9782)**	***P***
**Patients with coronary anomaly**			
Total number	**138**	**203**	
Prevalence	7.85%	2.08%	<0.01
Age in years (mean±SD)	56.4±14.1	68.6±12.7	<0.01
Male gender	89 (64.5%)	142 (70.0%)	0.35
**Coronary risk factors**			
Smoking	38 (27.5%)	55 (27.1%)	0.93
Hypertension	55 (39.3%)	126 (62.1%)	<0.01
Diabetes	12 (8.7%)	33 (16.3%)	0.06
Positive family history	38 (27.5%)	47 (23.2%)	0.43
Dyslipidemia	46 (32.9%)	83 (40.9%)	0.19
**Clinical symptoms**			
None	66 (47.8%)	31 (15.3%)	<0.01
Typical angina	31 (22.5%)	106 (52.2%)	<0.01
Atypical chest pain	24 (17.1%)	41 (20.2%)	0.61
Dyspnoea	12 (8.6%)	56 (27.6%)	<0.01
**MPI findings**			
MPI	47 (34.1%)	12 (5.9%)	<0.01
Ischemia due to coronary anomaly	1	0	0.85
Fixed perfusion defect	0	1	0.41
Ischemia due to CAD	13	6	0.02

CCTA=coronary computed tomography angiography, IAC=invasive coronary angiography, BMI=body mass index, HR= heart rate, MPI=myocardial perfusion imaging, CAD=coronary artery disease.

### Coronary anomalies

**Figure 1:**
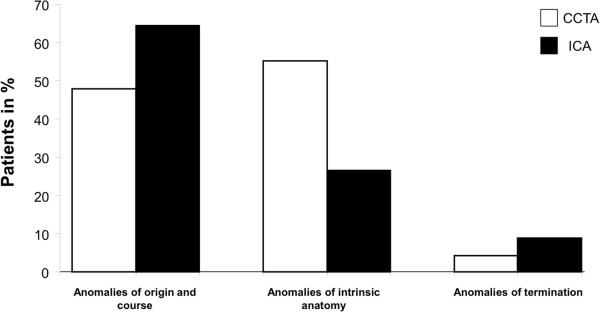
Percentage of patients of the three sub-groups of coronary anomalies identified with both imaging modalities (CCTA/white and ICA/black): 1) Anomalies of origination and course 2) Anomalies of intrinsic coronary artery anatomy 3) Anomalies of coronary termination.

All anomalies were classified into three main groups: [1] anomalies of origin and course, with a prevalence of 47.8% (n = 66; CCTA) and 64.5% (n = 131; ICA; p < 0.01), respectively. [2] Anomalies of intrinsic coronary arterial anatomy were found in 55.1% (n = 76) of the patients by CCTA, but only in 26.6% (n = 54 by ICA; p < 0.01). [3] Anomalies of coronary termination were detected in 4.3% (n = 6) of the patients by CCTA and in 8.9% (n = 18) by ICA (Table 2 and Figure 1; p = n.s.).

**Table 2:** Patients with coronary anomalies

	**CCTA (n=138)**	**ICA (n=203)**	***P***
**1. Anomalies of origination and course**	**66 (47.8%)**	**131 (64.5%)**	**<0.01**
**1.1 Absent left main trunk (split origination of LMA)**	16 (11.6%)	73 (36.0%)	<0.01
**1.2 Anomalous location of coronary ostium within aortic root or near proper aortic sinus of Valsalva**	1 (0.7%)	0 (0%)	0.84
**1.3 Anomalous location of coronary ostium outside normal coronary aortic sinuses**			
LCA arising from posterior facing sinus (ALCAPA)	2 (1.5%)	0 (0%)	0.32
RCA arising from anterior right facing sinus	3 (2.2%)	32 (15.8%)	<0.01
**1.4 Anomalous origination of coronary ostium from opposite, facing “coronary” sinus (which may involve joint origination or adjacent double ostia)**			
**1.4.1 RCA arising from left sinus with anomalous course**	11 (8.0%)	13 (6.4%)	0.73
Between aorta and pulmonary artery	11 (8.0%)	n.a.	-
**1.4.2 LAD arising from right anterior sinus. with anomalous course**	4 (2.9%)	7 (3.5%)	0.78
Between aorta and pulmonary artery	2 (1.5%)	n.a.	-
Anterior to pulmonary outflow or precardiac	1 (0.7%)	n.a.	-
Postero anterior interventricular groove	1 (0.7%)	n.a.	-
**1.4.3 Cx arising from right anterior sinus. with anomalous course**	19 (13.8%)	5 (2.5%)	<0.01
Retroaortic	19 (13.8%)	n.a.	-
**1.5 Single coronary artery**	4 (2.9%)	0 (0%)	0.05
**1.6 Bland-White-Garland Syndrome**	2 (1.5%)	1 (0.5%)	0.74
**2. Anomalies of intrinsic coronary arterial anatomy;**	**76 (55.1%)**	**54 (26.6%)**	**<0.01**
Absent coronary artery	4 (2.9%)	n.a.	-
Coronary hypoplasia	11 (8.0%)	n.a.	-
Intramural coronary artery (myocardial bridge)	59 (42.8%)	43 (21.2%)	<0.01
Double RCA	2 (1.5%)	2 (1.0%)	0.70
Double LAD	0 (0%)	9 (4.4%)	0.03
**3. Anomalies of coronary termination, Fistulas from RCA, LCA or infundibular artery to:**	**6 (4.3%)**	**18 (8.9%)**	**0.17**
Superior vena cava	2 (1.5%)	0 (0%)	0.32
Pulmonary artery	1 (0.7%)	8 (3.9%)	0.14
Pulmonary vein	3 (2.17%)	0 (0%)	0.13
Right atrium	0 (0%)	2 (1.0%)	0.65
Right ventricle	0 (0%)	2 (1.0%)	0.65
Left atrium	0 (0%)	1 (0.5%)	0.41
Left ventricle	0 (0%)	5 (2.5%)	0.16

CCTA=coronary computed tomography angiography, IAC=invasive coronary angiography, LMA=left main coronary artery, LCA=left coronary artery, LAD=left anterior descending artery, RCA=right coronary artery, Cx=circumflex coronary artery.

#### Anomalies of origin and course

**Figure 2:**
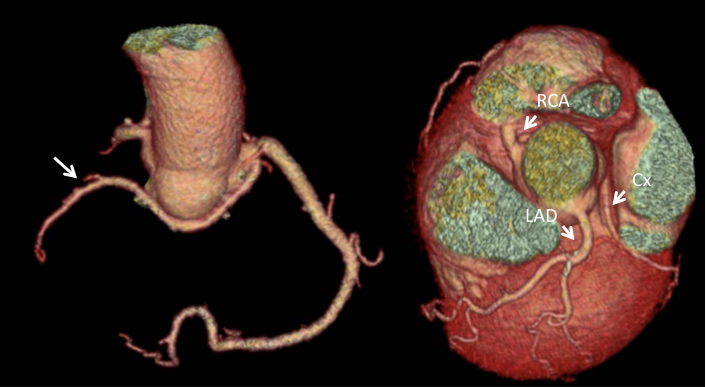
Volume-rendered three-dimensional reconstructions of the coronary tree and heart by CT coronary angiography in a patient presenting with previous syncope showing the circumflex artery arising from the right anterior sinus with anomalous retroaortic course.

In the ICA group, an absent left main trunk was the most frequently observed anomaly occurring in 36.0% (n = 73) of the patients, while by CCTA such a finding occurred in only 11.6% (n = 16); p < 0.01. In the CCTA group, LCX arising from the right anterior sinus, with anomalous retroaortic course (Figure 2) was noted most commonly (13.8%; n = 19), while in the ICA group this anomaly was a rare finding (2.5%; n = 5; p < 0.01).

**Figure 3:**
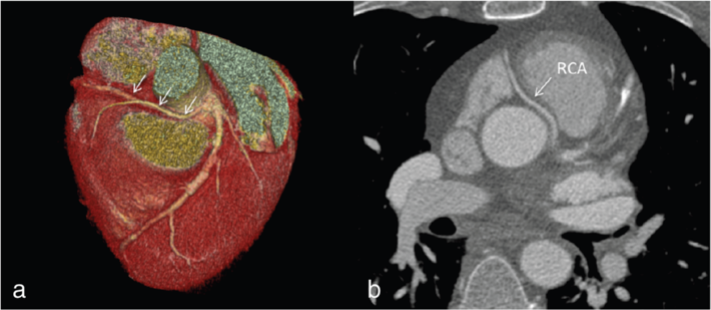
**A patient with an anomalous origination of the right coronary artery (RCA). (a)** Volume-rendered three-dimensional image showing that RCA is arising from the left coronary sinus with anomalous course between aorta and pulmonary trunk. **(b)** Axial images of the same patient showing the potentially malignant course of the RCA.

**Figure 4:**
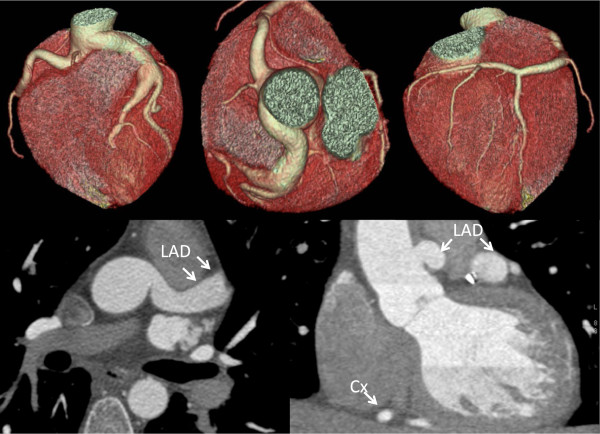
**A patient with bland-white-garland syndrome (anomalous origin of the left coronary artery arising from the pulmonary artery) after cardiac surgery and replacement of origination.** Volume-rendered images reveal now, corrected origination of the left coronary artery from the aorta with massive dilatation of the main stem (14.0 mm) and LAD (11.4 mm).

A potentially serious or malignant coronary anomaly was defined as a course of the coronary artery between aorta and pulmonary artery [16]. Obviously, this could only be detected by CCTA imaging (ICA non-accessible; Figure 3). A potentially serious coronary anomaly was found in 9.4% (n = 13) of the patients by CCTA including 8.0% (n = 11) of the patients with a RCA arising from left sinus and 1.5% (n = 2) of the patients with a LAD arising from the opposite sinus, both coursing between the aorta and pulmonary artery. We also included patients with a Bland-White-Garland Syndrome (anomalous origin of the left coronary artery arising from the pulmonary artery) into this group which was detected in 1.5% (n = 2) by CCTA (Figure 4) and in 0.5% (n = 1) by ICA (p = n.s.) Table 2.

#### Anomalies of intrinsic coronary arterial anatomy

**Figure 5:**
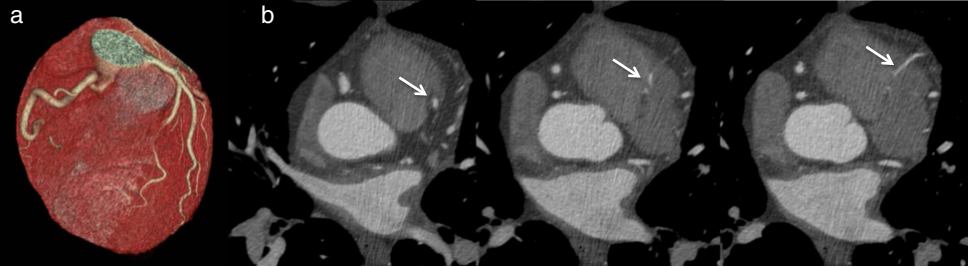
**(a) Volume-rendered reconstruction showing myocardial bridge of the left anterior descending artery in a 65-year-old man who was referred for the exclusion of coronary artery disease. (b)** Axial images revealing myocardial bridge with intramuscular course of the proximal left anterior descending artery between the left ventricular muscles.

**Figure 6:**
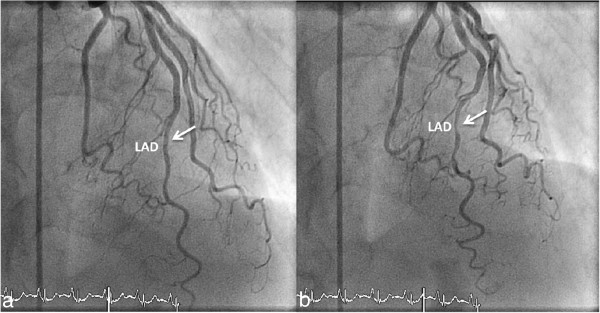
**A patient with angina pectoris underwent coronary angiography.** Images reveal myocardial bridging in the mid LAD **(a)** during diastole and **(b)** during systole.

The prevalence of an intramural coronary artery (myocardial bridge) was the most common finding with both imaging modalities; however, more cases of myocardial bridging were identified by CCTA (42.8%; n = 59) than with ICA (21.2%; n = 43; (p < 0.01) (Figures 5 and 6). In the CCTA as well as in the ICA group the majority of myocardial bridges were found in the LAD (n = 52/59 vs. n = 42/43) with the predominant location of the middle segments of the vessel.

#### Coronary anomalies of termination

**Figure 7:**
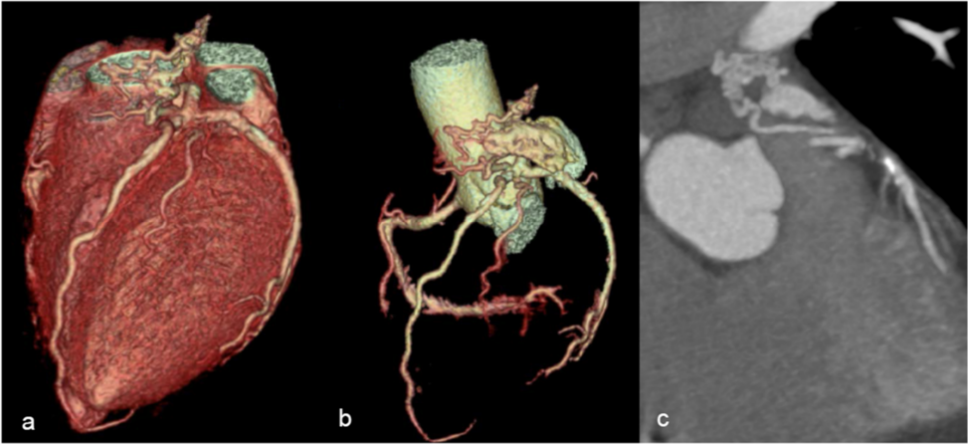
**A patient with a complex coronary-pulmonary artery fistula presenting with previous syncope. (a, b)** Volume-rendered three-dimensional images show a big plexus of the tortuous vessels arising from the proximal left anterior descending artery. **(c)** Axial images of the same patient show the course of the fistula ending into a pulmonary vein.

**Figure 8:**
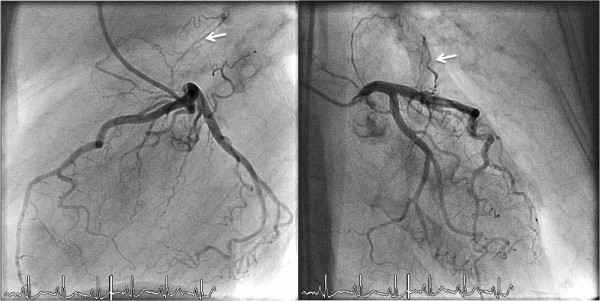
A patient with angina symptoms during exercise stress test revealed a rudimentary fistula from the LAD to the pulmonary artery.

**Figure 9:**
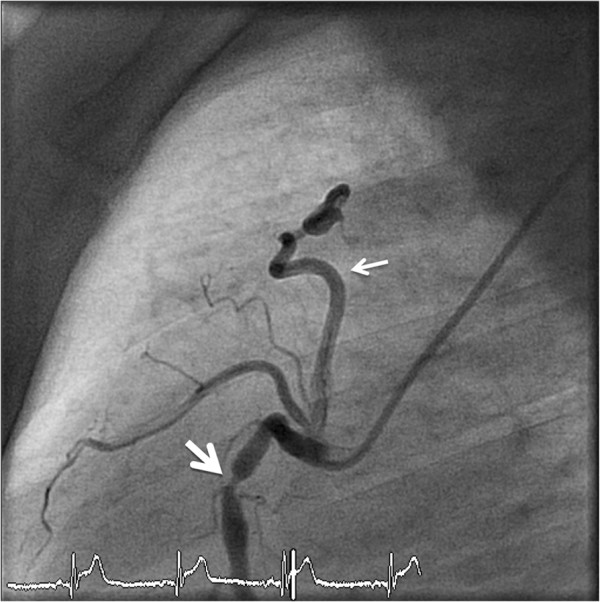
**A patient underwent coronary angiography due to inferior ST-segment elevation myocardial infarction.** Coronary images revealed AV-Fistula from the proximal RCA to the pulmonary artery. The (bold arrow) shows the culprit lesion in the mid RCA.

Anomalies of coronary termination were most commonly fistulas originating from the RCA, LCA or infundibular artery (Figure 6). In fact, this group showed the lowest frequency of occurrence. Fistulas either terminated into the pulmonary artery (Figures 7, 8 and 9) in 0.7% (n = 1) of the patients by CTCA and 3.9% (n = 8) by ICA (p = n.s.) or into the pulmonary vein in 2.2% (n = 3) by CTCA, while no patient within the ICA group were identified to have such an anomaly.

### Myocardial perfusion imaging

Myocardial Perfusion Imaging (MPI) was performed in 47 patients (34.1%) of the CCTA group and in 12 patients (5.9%) of the ICA group (p < 0.01). Despite the high rate of a potentially malignant course of the coronary arteries of 9.4% in the CTCA group, no ischemia could be attributed to the anomaly itself. However, in one patient out of the entire group of patients with a coronary anomaly detected by CCTA myocardial ischemia was noted. No patient of the ICA group that had undergone myocardial perfusion imaging due to coronary anomaly 5.9% (n = 12) exhibited any perfusion defect (Table 1).

### Radiation exposure

The radiation exposure from CCTA averaged DLP 133.4 ± 55.6 mGy×cm^2^ resulting in 1.9 ± 0.7 mSv. Estimation of the radiation exposure of patients undergoing coronary angiography was limited (due to a separate reporting system). Therefore we assessed the radiation exposure in a random sample of 34 patients out of the 203. The mean DAP was 86.6 ± 55.4 mGy×cm^2^ resulting in a mean radiation dose of 19.1 ± 12.2 mSv.

### Invasive procedures in the CCTA group

Among 138 patients demonstrating a coronary anomaly by CCTA, 22% (n = 30) underwent an additional invasive evaluation and eventually in 12.3% (n = 17) significant coronary artery disease was documented, while the remaining patients (9.4%; n = 13) showed no evidence of obstructive coronary artery disease at ICA.

## Discussion

Up until recently, ICA has been considered the gold standard for the evaluation of coronary anomalies. Meanwhile CCTA has emerged as a non-invasive alternative for the evaluation of the coronary architecture, particularly after introduction of modern protocols allowing to perform CCTA with radiation doses substantially lower than that from ICA with improved spatial resolution [17]. We hypothesized that the number of coronary anomalies by CCTA may more closely reflect their real prevalence in the general population. The reported incidences of coronary anomalies were mostly gained by ICA [18], in which the referral threshold is assumedly higher than for CCTA. Indeed, the number of anomalies identified in our survey by CCTA was remarkably higher compared to previous studies [19,20]. However, some subgroups of patients underwent CCTA (for example young patients) because of the clinical suspicion of coronary anomalies, while ICA was performed especially for CAD. This issue might partially explain the higher rates of coronary anomalies discovered by CCTA creating a selection bias. Nevertheless the overall prevalence of coronary anomalies detected by CCTA was significantly higher than by coronary angiography (7.85% versus 2.02%; p < 0.01). In this regard, it has to be stated, that the prevalence is also highly dependent on the definition of coronary anomalies. One of the largest clinical data sets which was reported by the Cleveland Clinic Foundation, observed coronary anomalies in 1.3% (n = 1′461) out of 126′595 investigated patients undergoing ICA [21].

In the present study, coronary anomalies were divided into two groups, i.e. anomalies of the origin/distribution and anomalies of termination. The clinically most relevant coronary anomalies are those with an anomalous course between the large intra-thoracic vessels (aorta and pulmonary artery). In our study, anomalies coursing between the aorta and the pulmonary trunk showed a prevalence of 9.4% by CCTA, which represents a higher occurrence compared to former studies, primarily based on autopsy data [22,23]. Of note, a discrimination of the course of a potentially malignant anomaly is only of reliable accuracy by CCTA, while ICA is limited by its 2-dimensional projections. Notably, none of our patients with an abnormal course revealed any perfusion defects by myocardial perfusion imaging. At our institution stress testing is usually performed with adenosine unless contraindicated. In this regard some of our patients (n = 5/13) with potentially serious coronary anomalies in the CCTA group were stressed with adenosine, although exercise testing is more physiological as the coronary artery may be squeezed between the pulmonary artery trunc and the aorta. However, these patients were usually referred for a combined investigation with CCTA and SPECT, and incidentally diagnosed with a potentially serious coronary anomaly after the SPECT results. Several studies have reported an increased risk for sudden cardiac death [24] as such patients showing myocardial ischemia [25]. However, only limited data are available concerning the long-term outcome of such patients without demonstrable myocardial ischemia [3,26]. Certainly, asymptomatic patients without stress-induced ischemia remain a clinical challenge. There is still controversy concerning the management of these patients. While some favor a surgical approach others recommend a conservative management. Since sudden cardiac death is associated with this anomaly related to severe exercise, patients are recommended to avoid extreme exertion and should be followed medically.

Beside the potential to describe the exact course of aberrant coronary vessels [27], CCTA offers another important advantage: Radiation dose exposure can be reduced to very low levels by applying novel CT protocols such as prospective ECG-triggering [6-9,28], tube current modulation [29] or high-pitch modus [7]. In addition several studies have demonstrated superiority of CCTA over ICA [30] in detecting coronary anomalies leading to a 100% identification rate of anomalies when analyzed with CCTA compared to 53% with ICA in a small series of 16 patients [31]. CCTA has recently been advocated as first-line method for known or suspected anomalies by the American College of Cardiology, provided appropriate criteria are used [32].

Interestingly, the most common finding in the CCTA group was myocardial bridging occurring in 43%, while such an anomaly was found in only 21% by ICA. Previous studies have reported myocardial bridging in 1.5-16% when assessed by ICA, while a substantially higher number up to 80% was observed in autopsy series [33]. Thus, myocardial bridging is a very common finding at autopsy even in normal subjects, but is considered clinically significant only when associated with chest pain and myocardial ischemia [34,35]. In our CCTA patient population, none of the patients with myocardial bridging revealed any perfusion defect in myocardial perfusion studies.

A further very common finding with both imaging modalities was the absence of the left main artery ranging between 12% by CCTA to 36% by ICA. These results are in line with Yamanaka et al. who found this anomaly to be most common (30.4%) among 126′595 patients evaluated by ICA. The prevalence among the general population is considered to be 0.41% [21]. This anomaly is usually regarded as benign, since the distribution of the vessels is normal.

This was a retrospective analysis and not a head to head comparison between two different techniques. Indeed, only a few patients underwent both imaging modalities. Furthermore this study did not include a randomly assigned study population rather than patients who were primarily referred by cardiologists for evaluation of suspected CAD or coronary anomaly which could have attributed to the higher identification of coronary anomalies among CCTA. Finally, young patients are more likely to be referred to a non-invasive imaging study with CCTA than for ICA, which may have introduced a potential selection bias.

## Conclusion

Our study indicates that coronary anomalies can be detected with both imaging modalities; however CCTA is superior in identifying the course of potentially malignant anomalies. The prevalence of coronary anomalies is substantially higher on CCTA than ICA even after exclusion of patients with myocardial bridging which is more frequently found with CCTA. This suggests that the true prevalence of coronary anomalies in the general population may have been underestimated based on ICA.

## Abbreviations

CAD: Coronary artery disease; CCTA: Coronary computed tomography angiography; CT: Computer tomography; ICA: Intracoronary angiography.

## Competing interests

The authors declare that they have no competing interest.

## Authors’ contributions

All authors have substantially contributed to the submitted work. All authors read and approved the final manuscript.

## Pre-publication history

The pre-publication history for this paper can be accessed here:

http://www.biomedcentral.com/1471-2261/14/81/prepub
